# Reaction Times among Batik Workers: The Influence of Gender and Occupational Lead Exposure

**DOI:** 10.3390/ijerph182312605

**Published:** 2021-11-30

**Authors:** Denny Agustiningsih, Meida Sofyana, Santosa Budiharjo, Sri Awalia Febriana, Hikmawati Nurokhmanti, Suhartini Suhartini, Dewanto Yusuf Priyambodo, Dwi Aris Agung Nugrahaningsih, Roto Roto, Rakhmat Ari Wibowo

**Affiliations:** 1 Department of Physiology, Faculty of Medicine, Public Health and Nursing, Universitas Gadjah Mada, Yogyakarta 55281, Indonesia; denny_agustiningsih@ugm.ac.id (D.A.); meidasofyana@mail.ugm.ac.id (M.S.); 2 Department of Anatomy, Faculty of Medicine, Public Health and Nursing, Universitas Gadjah Mada, Yogyakarta 55281, Indonesia; santosabudiharjo@ugm.ac.id; 3 Department of Dermatology and Venereology, Faculty of Medicine, Public Health and Nursing, Universitas Gadjah Mada, Dr. Sardjito General Hospital, Yogyakarta 55281, Indonesia; awalia_febriana@ugm.ac.id; 4 Department of Medical Education and Bioethics, Faculty of Medicine, Public Health and Nursing, Universitas Gadjah Mada, Yogyakarta 55281, Indonesia; drhikma@ugm.ac.id; 5 Department of Forensic and Medicolegal, Faculty of Medicine, Public Health and Nursing, Universitas Gadjah Mada, Yogyakarta 55281, Indonesia; suhartini@ugm.ac.id (S.S.); dewanto.y@mail.ugm.ac.id (D.Y.P.); 6 Department of Pharmacology and Therapy, Faculty of Medicine, Public Health and Nursing, Universitas Gadjah Mada, Yogyakarta 55281, Indonesia; dwi.aris.a@ugm.ac.id; 7 Department of Chemistry, Universitas Gadjah Mada, Yogyakarta 55281, Indonesia; roto05@ugm.ac.id

**Keywords:** batik industries, neurocognitive performance, occupational health, occupational lead exposure, simple reaction time

## Abstract

(1) Background: Neglected occupational health and safety aspects in batik industries cause their workers to have an increased risk of lead exposure. The effect of occupational lead exposure on neurocognitive performance is inconclusive. Therefore, we conducted an observational study to examine the difference in simple reaction time between lead-exposed batik workers and non-exposed referents. (2) Methods: This cross-sectional study was conducted in seven batik enterprises in Lendah District, Indonesia, excluding workers with medical conditions impairing reaction time. Simple reaction time tests were conducted using an online tool. Two-way model ANCOVAs examined interactions between gender and job types on the mean differences in reaction time. (3) Results: After controlling for age and body mass index, we observed longer reaction times among lead-exposed batik workers than non-exposed referents with an adjusted mean difference of 0.19 (95% CI: 0.016–0.368) seconds. A more prominent detrimental effect of lead exposure on reaction time among female workers than among male workers was observed. (4) Conclusions: Our results suggest that occupational lead exposure could contribute to longer reaction time, notably among female workers. Thus, occupational health and safety precautions are vital to protect batik workers and preserve their important contributions to cultural heritage.

## 1. Introduction

Batik has been recognized as an intangible cultural heritage of Indonesia [1]. In addition to its cultural contribution, batik industries are artistic industries contributing to Indonesia’s gross domestic product of more than 100,000 trillion rupiahs and employing more than 5 million persons annually [2]. Furthermore, while there was a huge economic decline during the COVID-19 pandemic, batik industries still contribute to maintaining Indonesian profit-making by expanding their export value [3].

The considerable contribution of batik industries to the economic sector is not followed by attention to these industries’ occupational health and safety aspects. Several factors can potentially harm batik workers’ health, including lead content in dyes, the un-ergonomic position at work, and non-standard personal protective equipment. Previous studies found that workers of the batik industry are prone to occupational chemical hazards [4,5]. Batik dye contains several toxic heavy metal elements, including cadmium, lead, arsenic, cobalt, and several essential elements required for maintaining human physiological functions, including zinc, chromium, and copper [6,7]. Unfortunately, our prior study on air pollutants in batik workplaces in Lendah, Yogyakarta, found that the lead concentration exceeded occupational health standards [7]. Lack of regulation and monitoring of lead exposure and unsafe work attitudes could contribute to chronic lead exposure among batik workers [4,8]. Chronic lead exposure among these vulnerable populations could result in a vicious cycle between lead exposure and poor economic status as well as poor educational attainment [9].

Both short- and long-term exposures to lead can cause serious health problems [10]. Lead poisoning often goes unnoticed because the symptoms are nonspecific and can occur slowly [11]. High levels of lead exposure can cause anemia, weakness, kidney damage, liver damage, cardiovascular disease, and even neurocognitive disorders [10,12,13,14]. While very high lead exposure can cause death, chronic low-level lead exposure could be associated with an increased risk of all-cause mortality [15].

Several systematic reviews suggested the detrimental effect of occupational lead exposure resulting in blood lead levels of lower than 70 µg/dl on neurocognitive performance [16,17]. However, their results were inconclusive. In addition, a recent study found that two years of same-level occupational lead exposure did not cause significant cognitive decline, but the statistically insignificant findings caused by insufficient sample size in that study could not be overlooked [18].

People exposed to lead often show impaired performance on a neurobehavioral test involving attention, processing, speed, visuospatial abilities, working memory, and motor function [19]. A simple test to observe neurobehavioral performance is a visual reaction time test. Although categorized as an unsophisticated evaluation tool, its validity and reliability are acceptable for monitoring changes in cognitive capacity by either pharmacological or non-pharmacological interventions in a productive work environment [20,21,22]. Therefore, we conducted an observational study to examine the difference in reaction time between batik workers with lead exposure and non-exposure day jobs.

## 2. Materials and Methods

### 2.1. Study Design and Setting

An observational cross-sectional study was conducted at seven batik enterprises in Lendah District, Yogyakarta, Indonesia, from July–October 2020 in compliance with the Declaration of Helsinki. The study registry was approved by the Medical and Health Research Ethics Committee, Faculty of Medicine, Public Health and Nursing, Universitas Gadjah Mada. The study protocol was written according to the Strengthening the Reporting of Observational Studies in Epidemiology (STROBE) statement [23].

### 2.2. Participants

To be eligible for inclusion, adult participants (18–60 years old) had to be registered as workers at the batik enterprises, and conveniently agreed to be respondents. Participants were excluded if they had one of the following conditions: consumed alcohol within 24 h [24], hypertension [25,26], type 2 diabetes mellitus [27], liver disease [28], renal disease [29], heart failure [30], and anemia [31], since those conditions could impair reaction time. Exclusion processes were conducted by history-taking, physical examination, and laboratory examination conducted by trained general practitioners. Older adults aged 60 or over were excluded because of their intraindividual reaction time performance [32]. Participant’s jobs were categorized as lead-exposed occupations if they reported conducting canting (a traditional hand-drawing method on fabric using liquid wax and dye), dipping the fabric in the dye, and washing the fabric after the coloring process because our prior study found excessive lead concentration in batik workers’ inhaled air during these processes at the workplaces [7]. A previous study found that participants who had these jobs were found to have elevated blood lead levels because they were exposed to synthetic dye-containing lead [33].

### 2.3. Outcome Measures

Simple reaction time was measured using online tools on preferred hands [34]. Participants were given five tests as the familiarization process prior to measurement. The mean of the five measurements was recorded as the reaction time. Research staff who conducted simple reaction time measurements were blinded from participants’ job types and Nordic scores to minimize observer bias.

Body mass index (BMI) and Nordic score were measured as potential confounders [35,36,37,38,39,40,41]. BMI was calculated by taking participants’ weight and height. Weight was measured using a standard calibrated scale (©SECA, Hamburg, Germany), and height was measured using a stadiometer (©SECA, Hamburg, Germany). The mean of three measurements of weight and height were recorded. The Nordic score was measured by Nordic Body Map questionnaire. The Nordic Body Map questionnaire is a standardized questionnaire to measure complaints (pain, tenderness, and stiffness) in the body during work performed in the recent three months. The Nordic score was categorized into seven segmental scores: neck, shoulder, back, right upper limb, left upper limb, right lower limb, and left lower limb [41].

Lead concentrations were analyzed using atomic absorption spectrophotometry (AAS) from conveniently collected venous whole blood and urine samples. Five milliliters of whole blood samples from each respondent were frozen at −4 °C, and five milliliters of urinary samples were stored with the addition of HNO_3_. Due to the pandemic, the AASs were conducted in the first week of November 2021 with the lowest threshold for lead detected at 0.01 ppm.

### 2.4. Sample Size Considerations

A sample size calculation to detect the main effect of lead-exposed job and potential confounding interaction on the reaction time as a primary outcome was conducted using G*Power analysis [42]. A minimum sample of 244 was calculated using a power of 0.8, an alpha level of 0.05, four covariates (age, BMI, years of service, and Nordic score), and an effect size of 0.2 [16]. Because of the low population in small enterprises, we anticipated the limited number of participants by conducting a post-hoc power analysis since we did not reach the minimum sample size.

### 2.5. Statistical Analysis

Descriptive statistics and mean or median difference between sex, level of education, and job status were used to report participants’ baseline characteristics, including age, BMI, Nordic score, and years of service. Reaction time was presented utilizing box plots based on gender, level of education, and job type. Then, Spearman correlation analysis was conducted to measure the correlation between age, Nordic score, years of service, and reaction time since the data was skewed. Pearson correlation analysis was conducted to measure the correlation between BMI and reaction time. Variables with statistically significant correlation using cut-off *p*-values of 0.25 were used as covariates in the analysis of covariance (ANCOVA) to control confounders [43,44]. Two-way model ANCOVAs were conducted to examine the interaction between gender and job type on the mean differences in reaction time since the residuals were normally distributed [45].

## 3. Results

Among the 250 registered workers in seven small enterprises in Lendah District, Indonesia, 100 workers voluntarily agreed to participate. We excluded 39 participants: four subjects with anemia (two male subjects and two female subjects with average hemoglobin concentration of 11.8 mg/dl and 11.6 mg/dl, respectively), nine subjects were elderly, and 18 subjects with abnormal liver function tests. Among the 59 eligible respondents, ten subjects did not attend the reaction time measurement. Thus, this study’s recruitment, eligibility, and completion rates were 40%, 69%, and 85%, respectively. Eight subjects were excluded from the analysis because they had outlier values for reaction time. A post-hoc power analysis showed that the power of our study was 58%.

Our respondents had already been working in this industry for a median of 5 years (Table 1). They did not have an undergraduate degree. Most of them were female. Male workers were younger, had less body mass index, had more musculoskeletal complaints, and had fewer years of service. Four of twenty-one male workers were not smokers. There were 15 urinary samples conveniently collected from 52 workers who were exposed to lead during their work. Among them, eight samples contained lead below our machine threshold detection, and seven samples contained lead with an average of 11.5 µg/dl (95% confidence interval (CI): 8.2 to 14.7 µg/dl). Two urinary samples were collected among seven workers who were not exposed to occupational lead, and none of them contained lead beyond the detection threshold. None of the 59 blood samples contained lead beyond the detection threshold. While differences in age, BMI, and several musculoskeletal complaints between genders were statistically significant, differences in the years of service were only marginally significant. We found neither differences in age, BMI, musculoskeletal complaints, nor years of service between lead exposure status.

**Figure 1:**
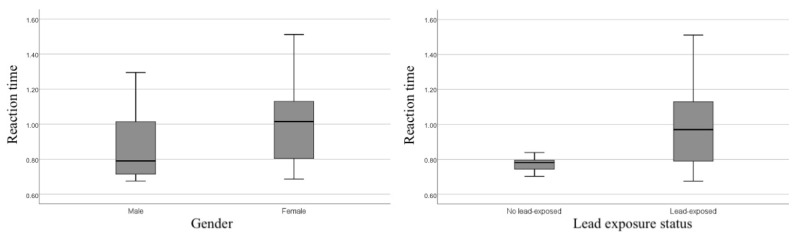
Reaction time by gender and lead exposure status.

We found significant differences in reaction time between gender and lead-exposed status in independent t-tests (Figure 1). Male workers had 0.14 s faster reaction times than female workers (*p* = 0.02). In addition, we also observed faster reaction time among non-lead exposure workers with a mean difference of 0.19 s (*p* < 0.001).

Age and body mass index had weak positive correlations with reaction times (Table 2). Years of service and the Nordic scores had very weak correlations with the reaction time, but their correlation was not statistically significant. Thus, we only include age and body mass index as confounding factors in examining the difference of reaction time between lead-exposure and no lead-exposure jobs.

**Figure 2:**
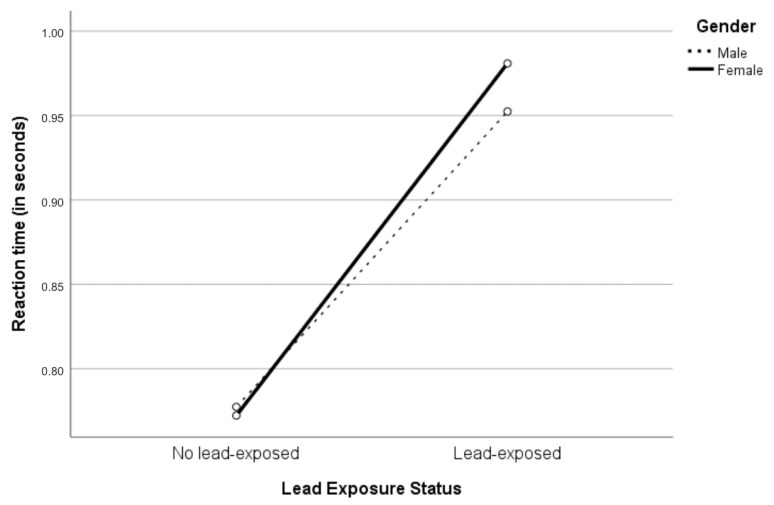
Interaction between gender and lead exposure status to the reaction time.

Since age and body mass index could impede the effect of lead exposure during work on reaction time, two-way ANCOVA was performed by controlling for age and body mass index (Table 3). A greater detrimental effect of lead exposure on reaction time among female workers than among male workers could be observed (Figure 2). However, two-way interaction between gender and lead-exposed status on reaction time could not be observed, F(1,45) = 0.036; *p* = 0.85, partial η2 = 0.001. While there was a statistically significant main effect of lead exposure status, F(1,45) = 4.820; *p* = 0.033, partial η2 = 0.097, the main effect of gender on reaction time could not be observed, F(1,45) = 0.018; *p* = 0.895, partial η2 = 0.000. The adjusted marginal mean reaction time of the no lead-exposed workers (0.775 s) was faster than the lead-exposed workers (0.967 s), resulting in a statistically significant difference of 0.192 (95% CI: 0.016 to 0.368) seconds.

## 4. Discussion

This study found a longer reaction time among lead-exposed batik workers than non-exposed batik workers. Our findings could add further support to previous studies reporting detrimental effects of occupational lead exposure captured in the recent systematic reviews [16,17]. While their overall metanalysis result was inconclusive, Goodman et al. suggested a significant difference of cognitive function examined by the digit symbol test, a more sensitive test than the simple reaction time, to detect a change in cognitive function [16,46]. Our results call for workplace intervention to protect batik workers from chronic lead exposure considering their contribution to cultural heritage.

The mechanism of chronic lead toxicity during adulthood in the cognitive domain has just started to be explored in detail [47]. Several previous studies have demonstrated the effect of occupational lead exposure with executive functions decline [48], slowed decision-making abilities, and reaction times [49]. A recent animal study found detrimental effects of chronic lead exposure on neurotransmitters in adult rats’ prefrontal cortex, representing chronic lead exposure during adulthood [50]. Lead can substitute for calcium in several regulatory events that involve calmodulin [51], interfering with energy metabolism and calcium release from mitochondria, resulting in priming activation of programmed cell death process [52,53]. These neurophysiological changes disrupt synaptic connectivity and neurogenesis, which have an essential role in neural plasticity. The most commonly used approach to monitoring neural plasticity is the measurement of reaction time (RT). Any increase in the RT to visual or auditory stimuli may indicate impairment or disruption in the cognitive processing, sensory information processing, or motor behavior initiation [54].

The mechanism of the detrimental effect of lead exposure during adulthood could be different from the effect of chronic lead exposure during prenatal development and childhood. During these early life phases, robust evidence has demonstrated detrimental effects of chronic lead exposure on hippocampus development and myelin synthesis during brain development [10,47,55] because lead disrupts the key molecules for neuronal migration and differentiation [56], decreasing the production of neuronal sialic acid for synapse formation [57], and premature differentiation of glial cells [58].

A more pronounced effect of lead exposure on reaction time among female workers than male workers might be assumed. Mansouri et al. also found different behavioral effects of chronic lead exposure between male and female rats [50]. Distinct hippocampus development and the differences in lead concentration and metabolism between male and female was suggested to be responsible for the sex-dependent effect of chronic lead toxicity on cognitive function [59]. On the contrary, a previous study found that men experienced a higher blood lead level than females in a similar occupational context [60]. While our study supports the gender-specific threshold of occupational lead exposure, the sex-dependent effect of chronic lead toxicity on reaction time needs further investigation.

Several studies suggested that musculoskeletal problems and complaints such as leg pain, low back pain, upper limb pain, and neck pain could result in delayed reaction time, but our study could not find a correlation between musculoskeletal complaints and reaction time [35,36,37,38,39,40]. In addition to the small sample size, no correlation between musculoskeletal problems and reaction time could be explained by the nature of the Nordic Body Map questionnaire, which assessed historical musculoskeletal pain complaints. When the light stimuli during the simple reaction time test arrive at the same time as the pain stimuli, faster processing of pain, rather than the processing of other stimuli by the human brain, could explain the dampening effect of pain on reaction time [61,62]. Consequently, battery tests of current pain complaints could be recommended for future research to control pain effects as confounding factors.

A previous systematic review found that most studies examining the correlation of occupational lead exposure and reaction time did not control premorbid states [16]. Our study tried to control the premorbid state by doing a comprehensive history-taking and physical examination by trained general practitioners and laboratory examination to exclude subjects based on excluding criteria. Nevertheless, by adjusting the result for age and BMI, our study still failed to control other interpersonal variabilities confounding the simple reaction time results. In addition to the strength of our study in controlling confounding factors, our study also informed recruitment, eligibility, and completion rates which will help plan future research.

A small sample size could be a limitation of our study. However, in our underpowered study, we could demonstrate a statistically significant main effect of lead exposure on reaction time. Thus, type II error should not be a matter of concern in our study. We also found that the average urinary lead concentration of workers exposed to lead during their work was close the upper limit of the guideline threshold [63]. However, we did not find lead concentration beyond the limit of detection in blood samples among our respondents. In addition to the variability of lead concentration in urine, this could be due to the instability of lead level in storage at −4 °C for more than 12 months [63,64]. Our study only measured blood and urinary lead level which represented acute lead intoxication and varied between individuals because of its dependence on kidney excretion and exposure time [18,65,66]. In the future, we could use lead bone level considered as cumulative lead storage in the bone during chronic lead exposure [18,65,66].

The influence of gender and lead exposure on reaction time in our study should be cautiously interpreted because of the cross-sectional design and confounding by co-exposures of tobacco smoking and other neurotoxic metals contained in batik dye, especially copper and cadmium [67,68,69,70]. Longitudinal studies on newly lead-exposed non-smoker workers are needed to establish the causal relationship between chronic lead exposure and reaction time. The level of education could moderately affect the correlation between age and health status on reaction time [71]. While our respondents did not have a college degree, findings from our study should not be directly generalized into other industries with college/university workers.

## 5. Conclusions

Our study contributes to the growing evidence of the effect of lead exposure during adulthood on the cognitive domain by showing a longer reaction time among lead-exposed batik workers than non-lead exposed workers. In addition to controlling for age, BMI, medical conditions, and other interpersonal variances which affect cognitive function, future longitudinal studies should utilize multiple cognitive function assessment tools and lead biomarkers. Workplace intervention considering health and safety precautions among batik workers should be implemented to protect them and to preserve their essential contributions to cultural heritage.

## Figures and Tables

**Table 1:** Characteristics of subjects.

	Total (M (SD)/Mdn (Min–Max)) N = 59	Male (M (SD)/Mdn (Min–Max)) N = 21	Female (M (SD)/Mdn (Min–Max)) N = 38	Difference (M Diff (*p*)/Mdn Diff (*p*))	Lead-Exposed (M (SD)/Mdn (Min–Max)) N = 52	No Lead-Exposed (M (SD)/Mdn (Min–Max)) N = 7	Difference (M Diff (*p*)/Mdn Diff (*p*))
Age (years)	40 (18–59)	25 (18–55)	44 (21–59)	**19.5 (*p* = 0.01)**	41 (18–59)	36 (24–45)	−1.5 (*p* = 0.506)
BMI (kg/m2)	22.45 (4.09)	20.75 (3.16)	23.64 (4.29)	**2.89 (*p* = 0.012)**	22.31 (4.18)	23.68 (3.17)	1.36 (*p* = 0.48)
Nordic score neck complaints	0 (0–4)	0 (0–3)	0 (0–4)	**0 (*p* = 0.019)**	0 (0–4)	0 (0–2)	0 (*p* = 0.615)
Nordic score shoulder complaints	0 (0–4)	1 (0–3)	0 (0–4)	**−1 (*p* = 0.022)**	0 (0–4)	2 (0–3)	2 (*p* = 0.206)
Nordic score back complaints	1 (0–8)	2 (0–8)	0.5 (0–8)	−1.5 (*p* = 0.052)	1 (0–8)	0 (0–3)	−1 (*p* = 0.313)
Nordic score right upper limb complaints	0 (0–10)	2 (0–10)	0 (0–9)	**−2 (*p* = 0.001)**	0 (0–10)	0 (0–4)	0 (*p* = 0.708)
Nordic score left upper limb complaints	0 (0–7)	0 (0–7)	0 (0–6)	0 (*p* = 0.194)	0 (0–7)	0 (0–0)	0 (*p* = 0.253)
Nordic score right lower limb complaints	3 (0–14)	0 (0–12)	0 (0–14)	0 (*p* = 0.185)	0 (0–14)	0 (0–12)	0 (*p* = 0.841)
Nordic score left lower limb complaints	0 (0–14)	0 (0–8)	0 (0–14)	0 (*p* = 0.145)	0 (0–14)	0 (0–8)	0 (*p* = 0.824)
Years of service (months)	60 (2–360)	60 (2–144)	84 (2–360)	24 (*p* = 0.053)	73 (2–360)	60 (5–144)	−13 (*p* = 0.634)

Bold font indicates statistical significance.

**Table 2:** Correlates of reaction time.

	Correlation Coefficient (*p*)
Age	0.47 (*p* < 0.001)
BMI	0.25 (*p* = 0.06)
Nordic score neck complaints	−0.092 (*p* = 0.52)
Nordic score shoulder complaints	−0.003 (*p* = 0.98)
Nordic score back complaints	0.059 (*p* = 0.68)
Nordic score right upper limb complaints	−0.189 (*p* = 0.18)
Nordic score left upper limb complaints	−0.055 (*p* = 0.70)
Nordic score right lower limb complaints	−0.018 (*p* = 0.89)
Nordic score left lower limb complaints	−0.027 (*p* = 0.85)
Years of service	0.158 (*p* = 0.27)

**Table 3:** Reaction time difference between gender and chemical exposure on occupation.

Reaction Time		No Lead-Exposed			Lead-Exposed	
	All (N = 7)	Male (N = 3)	Female (N = 4)	All (N = 52)	Male (N = 18)	Female (N = 34)
M	0.772	0.774	0.769	0.970 ***	0.888	1.023 ^+^
(SD)	0.052	0.068	0.036	0.206	0.192	0.201
Madj ^a^	0.775	0.777	0.772	0.967 *	0.952	0.981
(SE)	0.083	0.103	0.129	0.027	0.047	0.037

Note. Reaction time measured in seconds. ^a^ Adjusted for age and body mass index * *p* < 0.05 compared to no lead-exposed group, *** *p* < 0.001 compared to no lead-exposed group, ^+^ *p* < 0.05 compared to male.

## Data Availability

Not applicable.

## References

[1] United Nations Educational Scientific and Cultural Organization (2009). Subsidiary body for the examination of nominations to the representative list of the intangible cultural heritage of humanity. Convention for the Safeguarding of the Intangible Cultural Heritage.

[2] Vincent A. (2010). Batik industry of indonesia: The rise, fall and prospects. Stud. Bus. Econ..

[3] Kementerian Perindustrian Republik Indonesia (2020). Dilanda Pandemi, Ekspor Batik Indonesia Mampu Tembus USD 21,5 Juta. https://www.kemenperin.go.id/artikel/22039/Dilanda-Pandemi,-Ekspor-Batik-Indonesia-Mampu-Tembus-USD-21,5-Juta.

[4] Junaidi M.S., Fatoni R., Fatimah S. (2020). The Analysis of Occupational Safety and Health of the Batik Industry. Adv. Sustain. Sci. Eng. Technol..

[5] Nankongnab N., Silpasuwan P., Markkanen P., Kongtip P., Woskie S. (2015). Occupational Safety, Health, and Well-being Among Home-based Workers in the Informal Economy of Thailand. New Solut. A J. Environ. Occup. Health Policy.

[6] Soebaryo R.W. (2012). Batik Manufacturing Workers. Kanerva’s Occup. Dermatol..

[7] Febriana S.A., Ogiwati K., Tanziha I., Roto R., Sarian F.D., Prakoeswa C.R.S., Thursina C., Suhartini S., Vicaria L.D., Priyambodo D.Y. (2021). Initiating “Healthy Batik Village”/“Desa Batik Sehat” to empower batik workers through collaborative health, environmental and social interventions. Res. Sq..

[8] George Foundation Implementing a National Program in Developing Countries. Proceedings of the International Conference on Lead Poisoning, Prevention and Treatment.

[9] Obeng-Gyasi E. (2018). Lead Exposure and Oxidative Stress—A Life Course Approach in U.S. Adults. Toxics.

[10] Wani A.L., Ara A., Usmani J.A. (2015). Lead toxicity: A review. Interdiscip. Toxicol..

[11] Pagliuca A., Mufti G.J., Baldwin D., Lestas A.N., Wallis R.M., Bellingham A.J. (1990). Lead poisoning: Clinical, biochemical, and haematological aspects of a recent outbreak. J. Clin. Pathol..

[12] Can S., Bağci C., Ozaslan M., Bozkurt A.I., Cengiz B., Cakmak E.A., Kocabas R., Karadağ E., Tarakçioğlu M. (2008). Occupational lead exposure effect on liver functions and biochemical parameters. Acta Physiol. Hung..

[13] Harari F., Sallsten G., Christensson A., Petkovic M., Hedblad B., Forsgard N., Melander O., Nilsson P.M., Borné Y., Engström G. (2018). Blood Lead Levels and Decreased Kidney Function in a Population-Based Cohort. Am. J. Kidney Dis..

[14] Obeng-Gyasi E., Ferguson A., Stamatakis K., Province M. (2021). Combined Effect of Lead Exposure and Allostatic Load on Cardiovascular Disease Mortality—A Preliminary Study. Int. J. Environ. Res. Public Health.

[15] Lanphear B.P., Rauch S., Auinger P., Allen R.W., Hornung R.W. (2018). Low-level lead exposure and mortality in US adults: A population-based cohort study. Lancet Public Health.

[16] Goodman M., Laverda N., Clarke C., Foster E.D., Iannuzzi J., Mandel J. (2002). Neurobehavioural testing in workers occupationally exposed to lead: Systematic review and meta-analysis of publications. Occup. Environ. Med..

[17] Meyer-Baron M., Seeber A. (2002). A meta-analysis for neurobehavioural results due to occupational lead exposure with blood lead concentrations. Arch. Toxicol..

[18] Yu Y.-L., Thijs L., Saenen N., Melgarejo J.D., Wei D.-M., Yang W.-Y., Yu C.-G., Roels H.A., Nawrot T.S., Maestre G.E. (2020). Two-year neurocognitive responses to first occupational lead exposure. Scand. J. Work. Environ. Health.

[19] Fenga C., Gangemi S., Alibrandi A., Costa C., Micali E. (2016). Relationship between lead exposure and mild cognitive impairment. J. Prev. Med. Hyg..

[20] Burghart M., Craig J., Radel J., Huisinga J. (2019). Reliability and validity of a motion-based reaction time assessment using a mobile device. Appl. Neuropsychol. Adult.

[21] Laux R.C., Corazza S.T. (2019). Improvement of reaction time after a workplace physical activity intervention. Rev. Bras. De Med. Do Esporte.

[22] Richardson J.K., Eckner J.T., Kim H., Ashton-Miller J.A. (2020). A clinical method of evaluating simple reaction time and reaction accuracy is sensitive to a single dose of lorazepam. J. Psychopharmacol..

[23] Vandenbroucke J.P., von Elm E., Altman D.G., Gøtzsche P.C., Mulrow C.D., Pocock S.J., Poole C., Schlesselman J.J., Egger M. (2007). Strengthening the Reporting of Observational Studies in Epidemiology (STROBE). Epidemiology.

[24] Gunn C., Mackus M., Griffin C., Munafò M.R., Adams S. (2018). A systematic review of the next-day effects of heavy alcohol consumption on cognitive performance. Addiction.

[25] Edwards L., Ring C., McIntyre D., Carroll D., Martin U. (2007). Psychomotor speed in hypertension: Effects of reaction time components, stimulus modality, and phase of the cardiac cycle. Psychophysiology.

[26] Forte G., De Pascalis V., Favieri F., Casagrande M. (2019). Effects of Blood Pressure on Cognitive Performance: A Systematic Review. J. Clin. Med..

[27] Muhil M. (2014). Study of Auditory, Visual Reaction Time and Glycemic Control (HBA 1 C) in Chronic Type I I Diabetes Mellitus. J. Clin. Diagn. Res..

[28] Lauridsen M.M., Mikkelsen S., Svensson T., Holm J., Klüver C., Gram J., Vilstrup H., De Muckadell O.B.S. (2017). The continuous reaction time test for minimal hepatic encephalopathy validated by a randomized controlled multi-modal intervention—A pilot study. PLoS ONE.

[29] Brodski J., Rossell S.L., Castle D.J., Tan E.J. (2018). A Systematic Review of Cognitive Impairments Associated with Kidney Failure in Adults Before Natural Age-Related Changes. J. Int. Neuropsychol. Soc..

[30] Leto L., Feola M. (2014). Cognitive impairment in heart failure patients. J. Geriatr. Cardiol..

[31] Kahlon N., Gandhi A., Mondal S., Narayan S. (2012). Effect of iron deficiency anemia on audiovisual reaction time in adolescent girls. Indian J. Physiol. Pharmacol..

[32] Hultsch D.F., MacDonald S.W.S., Dixon R.A. (2002). Variability in Reaction Time Performance of Younger and Older Adults. J. Gerontol. Ser. B.

[33] Hastuti P., Sunarti S., Prasetyastuti P., Ngadikun N., Tasmini T., Rubi D.S., Sutarni S., Harahap I.K., Dananjoyo K., Suhartini S. (2018). Hubungan timbal dan krom pada pemakaian pewarna batik dengan kadar hemoglobin dan jumlah sel darah pada pengrajin batik Kecamatan Lendah Kulon Progo. J. Community Empower. Health.

[34] Allen J. (2002). The Online Reaction Time Test. https://faculty.washington.edu/chudler/java/redgreen.html.

[35] Kauranen K., Siira P., Vanharanta H. (2001). Delayed-onset muscle soreness and motor performance of the upper extremity. Graefe’s Arch. Clin. Exp. Ophthalmol..

[36] Nene A.S., Pazare P.A., Sharma K.D. (2012). A study of relation between body mass index and simple reaction time in healthy young females. Indian J. Physiol. Pharmacol..

[37] Sudheer C., Jagadeesan S., Kammar F.K. (2017). Impact of BMI on Visual Reaction Time in Individuals with BMI in Normal Range. Int. J. Physiol..

[38] Taimela S., Kujala U.M. (1992). Reaction Times with Reference to Musculoskeletal Complaints in Adolescence. Percept. Mot. Ski..

[39] Venna S., Hurri H., Alaranta H. (1994). Correlation between neurological leg deficits and reaction time of upper limbs among low-back pain patients. Scand. J. Rehabil. Med..

[40] Yassin M., Talebian S., Ebrahimi-Takamjani I., Maroufi N., Ahmadi A., Sarrafzadeh J., Emrani A. (2015). The effects of arm movement on reaction time in patients with latent and active upper trapezius myofascial trigger point. Med. J. Islam. Repub. Iran.

[41] Wilson J., Corlett E. (1991). Evaluation of human work: A practical ergonomics methodology. Appl. Ergon..

[42] Faul F., Erdfelder E., Lang A.-G., Buchner A. (2007). G*Power 3: A flexible statistical power analysis program for the social, behavioral, and biomedical sciences. Behav. Res. Methods.

[43] Bendel R.B., Afifi A.A. (1977). Comparison of Stopping Rules in Forward “Stepwise” Regression. J. Am. Stat. Assoc..

[44] Mickey R.M., Greenland S. (1989). The impact of confounder selection criteria on effect estimation. Am. J. Epidemiol..

[45] Blanca M.J., Alarcón R., Arnau J. (2017). Non-normal data: Is ANOVA still a valid option?. Psicothema.

[46] Jaeger J. (2018). Digit Symbol Substitution Test. J. Clin. Psychopharmacol..

[47] Sanders T., Liu Y., Buchner V., Tchounwou P.B. (2009). Neurotoxic Effects and Biomarkers of Lead Exposure: A Review. Rev. Environ. Health.

[48] Schwartz B.S., Stewart W.F., Bolla K.I., Simon D., Bandeen-Roche K., Gordon B., Links J.M., Todd A.C. (2000). Past adult lead exposure is associated with longitudinal decline in cognitive function. Neurology.

[49] Winneke G., Lilienthal H., Krämer U. (1996). The Neurobehavioural Toxicology and Teratology of Lead. Arch. Toxicol..

[50] Mansouri M.T., Naghizadeh B., López-Larrubia P., Cauli O. (2013). Behavioral deficits induced by lead exposure are accompanied by serotonergic and cholinergic alterations in the prefrontal cortex. Neurochem. Int..

[51] Goldstein G.W. (1993). Evidence that lead acts as a calcium substitute in second messenger metabolism. Neurotoxicology.

[52] Simons T.J.B. (1986). Cellular interactions between lead and calcium. Br. Med. Bull..

[53] Brookes P., Yoon Y., Robotham J.L., Anders M.W., Sheu S.-S. (2004). Calcium, ATP, and ROS: A mitochondrial love-hate triangle. Am. J. Physiol. Physiol..

[54] Latash M.L. (2008). Neurophysiological Basis of Movement.

[55] Pocock S.J., Smith M., Baghurst P. (1994). Environmental lead and children’s intelligence: A systematic review of the epidemiological evidence. BMJ.

[56] Silbergeld E.K. (1992). Mechanisms of lead neurotoxicity, or looking beyond the lamppost. FASEB J..

[57] Bressler J.P., Goldstein G.W. (1991). Mechanisms of lead neurotoxicity. Biochem. Pharmacol..

[58] Cookman G.R., King W., Regan C.M. (1987). Chronic Low-Level Lead Exposure Impairs Embryonic to Adult Conversion of the Neural Cell Adhesion Molecule. J. Neurochem..

[59] Singh G., Singh V., Sobolewski M., Cory-Slechta D.A., Schneider J.S. (2018). Sex-Dependent Effects of Developmental Lead Exposure on the Brain. Front. Genet..

[60] Bonberg N., Pesch B., Ulrich N., Moebus S., Eisele L., Marr A., Arendt M., Jöckel K.-H., Brüning T., Weiss T. (2017). The distribution of blood concentrations of lead (Pb), cadmium (Cd), chromium (Cr) and manganese (Mn) in residents of the German Ruhr area and its potential association with occupational exposure in metal industry and/or other risk factors. Int. J. Hyg. Environ. Health.

[61] Attridge N., Keogh E., Eccleston C. (2016). The effect of pain on task switching: Pain reduces accuracy and increases reaction times across multiple switching paradigms. Pain.

[62] Ploner M., Gross J., Timmermann L., Schnitzler A. (2006). Pain Processing Is Faster than Tactile Processing in the Human Brain. J. Neurosci..

[63] Center for Disease Control and Prevention (1994). Lead in Blood and Urine 8003. https://www.cdc.gov/niosh/docs/2003-154/pdfs/8003.pdf.

[64] Yang T., Sun C., Hsu H., Liou S., Wu D., Chu S., Tung H., Chen Y., Chen J. (2006). Stability of blood lead levels in stored specimens: Effects of storage time and temperature. J. Med. Sci..

[65] Barbosa F., Tanus-Santos J.E., Gerlach R.F., Parsons P.J. (2006). A critical review of biomarkers used for monitoring human exposure to lead: Advantages, limitations and future needs|uma avaliação crítica sobre biomarcadores utilizados para o monitoramento biológico de exposição ao chumbo: Vantagens, limitações e pers. Cienc. E Saude Coletiva.

[66] Shih R.A., Hu H., Weisskopf M.G., Schwartz B.S. (2007). Cumulative Lead Dose and Cognitive Function in Adults: A Review of Studies That Measured Both Blood Lead and Bone Lead. Environ. Health Perspect..

[67] Juliani A. (2021). Heavy metal characteristics of wastewater from batik industry in yogyakarta area, indonesia. Int. J. Geomate.

[68] Morgan S.F., Pickens R.W. (1982). Reaction time performance as a function of cigarette smoking procedure. Psychopharmacology.

[69] Pujol J., Fenoll R., Macià D., Martínez-Vilavella G., Alvarez-Pedrerol M., Rivas I., Forns J., Deus J., Blanco-Hinojo L., Querol X. (2016). Airborne copper exposure in school environments associated with poorer motor performance and altered basal ganglia. Brain Behav..

[70] Ciesielski T., Bellinger D.C., Schwartz J., Hauser R., Wright R. (2013). Associations between cadmium exposure and neurocognitive test scores in a cross-sectional study of US adults. Environ. Health.

[71] Tun P.A., Lachman M.E. (2008). Age differences in reaction time and attention in a national telephone sample of adults: Education, sex, and task complexity matter. Dev. Psychol..

